# The Mental Health Effects of Cumulative Lifetime Violence in Men: Disruptions in the Capacity to Connect with Others and Finding Ways to Reengage

**DOI:** 10.1177/23333936211021576

**Published:** 2021-06-16

**Authors:** Petrea Lea Taylor, Susanne O’Donnell, Judith Wuest, Kelly Scott-Storey, Charlene Vincent, Jeannie Malcom

**Affiliations:** 1 University of New Brunswick, Moncton, Canada; 2 University of New Brunswick, Fredericton, Canada

**Keywords:** interpretive description, qualitative methods, multiple methods, sequential design, violence, gender, men’s health, mental health, psychiatry, trauma and violence informed, Canada

## Abstract

We report qualitative findings of our Men’s Violence Gender and Health Study, a multiple method study using a sequential design in which we explored the mental health manifestations of cumulative lifetime violence in men. Survey results revealed that higher cumulative lifetime violence scores were significantly associated with higher scores on depression, post-traumatic stress disorder, and anxiety in a community sample of men (*n* = 685) living in Eastern Canada. To obtain a deeper understanding of men’s scores, we used an interpretive description approach to analyze data derived from 32 participant interviews. The main mental health manifestation of cumulative lifetime violence is *perceptual interference*, a sense of being disconnected or *detached* from others. This is managed by *rectifying detachment*, a process that includes efforts to gain connections with others. Findings suggest mental health needs in men with cumulative lifetime violence contradict gender role expectations to be stoic. Implications for nurses are explored.

## Introduction

In the recent launch of a special initiative to reduce mental health disparities, the World Health Organization (WHO) declared, “There can be no health or sustainable development without mental health” (WHO, 2019, p. 1). In general, mental health problems, particularly anxiety and mood disorders, are more prevalent among Canadian women than men; however, in comparison to women, men are three times more likely to report substance use disorder and three times more likely to die by suicide (Khan, 2017; Statistics Canada, 2019). This unique pattern of mental health, and the discrepancy between lower rates of depression and higher rates of suicide, are indicators of a silent crisis in men’s mental health (Affleck et al., 2018).

Commonly, men’s mental health issues are attributed to their attitudes, behaviors and choices, particularly those associated with dominant masculine gendered norms, while the contributions of other social determinants of health (SDOH) are ignored (Whitley, 2018). Violence is a SDOH that is pervasive throughout men’s lives and negatively influences men’s mental health (Haegerich & Hall, 2011; Scott-Storey et al., 2018; WHO, 2014). Historically, research in this field, has been limited to examining one or two types of violence at distinct points in the lifespan among either targets or perpetrators and has disregarded the cumulative effects of violence on health (Scott-Storey, 2011).

Moreover, little attention has been paid to how men themselves comprehend their mental health in the context of their lifetime experiences of violence. We addressed these knowledge gaps through analysis of qualitative interview data collected in the Men’s Violence Gender and Health Study (MVGHS) using an interpretive descriptive approach (Thorne, 2008). Specifically, we investigated men’s perceptions of the relationship between their experiences of cumulative lifetime violence and their mental health.

## Literature Review

Although violence is a global public health problem that results in “psychological harm” (WHO, 2002, p. 5), the scope and depth of mental health research among men with histories of violence is limited. In a critical review of the empirical evidence related to violence and women’s health, Scott-Storey (2011) concluded that adverse health effects can rarely be attributed to one type (physical, sexual, psychological) or form (intimate partner, familial, bullying, assault) of violence experienced as a single stage in life (childhood or adulthood) because multiple types and experiences of violence across the lifespan are common. Likewise, violence is most often defined narrowly in the literature, such as, child abuse, intimate partner violence (IPV), or workplace violence.

A large body of evidence supports the adverse effects of violence exposure in childhood violence on mental health in men and women; for example, experiences of child abuse in the home have been associated with anxiety disorders, depression, posttraumatic stress disorder (PTSD), substance use, and self-harming behaviors as adults (Cater et al., 2015; Copeland et al., 2018; Grigsby et al., 2020). Multiple studies demonstrate that experiencing IPV as a target is associated with depression, anxiety, PTSD, and suicide ideation in men (Bates, 2020a; Hines & Douglas, 2015; Machado et al., 2017; Próspero, 2007; Randle & Graham, 2011). Workplace violence exposure is also hazardous to men’s mental health; for instance, among police officers, witnessing violence incidents was a significant predictor of PTSD symptoms (Ménard & Arter, 2013) as was killing or significantly injuring someone early in their career (Komarovskaya et al., 2011). Mental health disorders including anxiety occur at higher rates in gang members and violent men in comparison to non-violent men (Coid et al., 2013) and depression occurs at higher levels in male teens who have experienced verbal bullying than those without bullying (Turner et al., 2013). Poor mental health was found to be more frequent in Swedish men aged 18 to 35 years who were the target of physical violence in the past year, the majority of which occurred in public places (Olofsson et al. 2009).

Evidence is also growing to support increased severity in mental health problems among those with multiple exposures to one or multiple types and/or contexts of violence as target and/or perpetrator. The association with symptoms of mental illness and experiencing violence as targets among men and women is stronger with multiple experiences in comparison to single violence events (Kilpatrick & Acierno, 2003; Simmons et al., 2015). More frequent community and interpersonal targeted violence has been correlated with more severe mental health problems in gay men living with HIV (Pantalone et al., 2010). Further, men who experienced violence as a target in both childhood and adulthood were more likely to meet the diagnostic criteria for anxiety disorders, depression, and PTSD than those with such experiences in adulthood only (Burns et al., 2016). As well, adult anxiety and depression (Thoresen et al., 2015), PTSD (Bender et al., 2015), and emotional distress scores (Hooven et al., 2012) increased with the number of different types of violence experienced in childhood.

Importantly, adverse childhood experiences that include several types of violence have a cumulative effect on adult violence experiences and mental health problems (Felitti et al., 1998; Hughes et al., 2017; Lee et al., 2020), especially in men (Choi et al., 2017). Moreover, greater severity of IPV as both target and perpetrator has been associated with greater risk for depression, PTSD, and suicidality in a study of African American men (Rhodes et al., 2009). Likewise, veterans who often experience recurring diverse violence as target and perpetrator in their combat and other service roles have been found to be at greater risk of developing depression, suicidal ideation (Blosnich et al., 2016; Bryan et al., 2018; Chapman et al., 2014), and PTSD (Veteran’s Affairs Canada, 2019).

Mental health is affected by allostatic overload and dysregulation of the body’s natural stress response system from violence-related recurrent stress causing physiological changes that lead to chronic mental health conditions including PTSD and depression (Keeshin et al., 2012; McEwen, 2012). But having a mental illness often increases the odds of experiencing violence as a target (Bhavsar et al., 2019) and perpetrator (Dorn et al., 2012; Trevillion et al., 2015). Shorey, et al. (2012) found that men who were convicted of IPV perpetration who met diagnostic criteria for depression, anxiety disorders, PTSD, or substance use disorders in comparison to those who did not reported significantly more severe patterns of partner aggression in the previous year. However, in general, those with mental illness are more likely to be a target than a perpetrator (Desmarais et al., 2014), with one in ten affected Canadians experiencing violence as targets within the past year (Burczycka, 2018). In summary, experiences of violence can lead to mental health problems that may increase the risk of subsequent violence as target and/or perpetrator, the cumulative effects of which may further compromise men’s mental health.

Yet there is a dearth of literature focusing on men’s perceptions of their lifetime experiences of violence and their mental health. Several qualitative studies have reported that feelings of insecurity and powerlessness related to childhood violence as targets in the family and/or community contributed to violence perpetration in adulthood (Ellis et al., 2017; Voith et al., 2020, Wei & Brackley, 2010). In a sample of men incarcerated for IPV, childhood experiences of abandonment, physical and sexual abuse, and witnessing IPV were viewed as *routine*; men described subsequent depression, anxiety and substance use in adolescence and problems in school with eventual involvement in the legal system (Watt & Scrandis, 2013). More attention has been paid to men’s perspectives as a target of IPV with consistent findings that societal and personal beliefs of gender roles and relationships influence men’s struggles to make sense of their experience and/or seek help to manage their responses such as fear, anger, shame and anxiety (Bates, 2020b; Brooks et al., 2020; Entilli & Cipolletta, 2017). Notably, in a community sample, mental health consequences of IPV for some men were found to be more impactful than physical as indicated by trying to kill oneself, and long-lasting PTSD that “destroyed my life and robbed me of a future.” (Bates, 2020a, p. 500).

In a study about workplace bullying by O’Donnell and MacIntosh (2016), gender beliefs also influenced mental health consequences of bullying at work and subsequent “abandonment” by workplace supports. Depression, anxiety, PTSD and/or substance use were associated with ongoing persistent stress, especially among those who believed men should be strong and self-reliant. Several men reported suicidal thoughts, making specific plans, with one reporting an incident of standing in front of a mirror with a knife to his throat. These qualitative studies provide richness and depth to understanding of how specific experiences of violence affect men’s mental health. However, no qualitative studies were found that considered men’s mental health in the context of their experiences of violence over their lifetimes.

Collectively, these quantitative and qualitative findings that multiple and/or recurring violence experiences across the lifespan have serious consequences for mental health; however, these studies are limited by neglecting the confounding effects of other experiences of lifetime violence not considered in the particular investigation. The literature search failed to reveal studies where violence was defined and/or measured comprehensively as cumulative lifetime violence. Data collected in our MVGHS provided an opportunity to address these gaps in knowledge.

## The Men’s Violence Gender and Health Study

In our MVGHS, a multiple method study using a sequential design, we used an online survey and qualitative interviews to explore how gender and cumulative lifetime violence (CLV) as target and/or perpetrator influence men’s health. Ethics approval was obtained from the affiliated university. Using online classified advertisements and posters in workplaces, a community convenience sample of 685 participants who self-identified as men between the ages of 19 to 65 years, English speaking and living in Eastern Canada was recruited from April 2016 to March 2018. Respondents received the letter of information and after providing online consent were directed to the online survey that included self-report questions focusing on socio-demographics, health behaviors, gender, common health problems, and lifetime violence experiences. Men who completed the online survey received an honorarium of $20 Canadian. As well, they were asked to provide contact information if they were interested in being interviewed about the study topics.

In the MVGHS study, CLV was measured comprehensively using 64 items about men’s physical, psychological and sexual violence experiences from childhood through adulthood, as target and/or perpetrator, in the context of gender, families, intimate relationships, schools, communities, and workplaces (Scott-Storey et al., 2020). Of this community sample of 685 men, 670 (97.8%) reported experiences of CLV as target and/or perpetrator. Mental health was measured using established and validated self-report measures; 46.5 % (*n* = 318) had scores consistent with problematic mental health symptomology (Table 1). Significant moderate or strong correlations were found between CLV scores and symptoms of depression (*r* = .494, *p* < .001), PTSD (*r* = .601, *p* < .001), and anxiety (*r* = .488, *p* < .001). These findings showed the direct association between CLV and mental health in this sample and supported the importance of developing a deeper contextual understanding these men’s experiences through analysis of MVGHS qualitative interview data.

**Table 1.** Indicators of Mental Health.

Scale	Full sample	Interview sample (*n* = 32)
^a^CESD-R Depression Indicator: *n* (%)	(*n* = 685)	
Yes (>15)	269 (39.3)	19 (59.4)
No (<16)	416 (60.7)	13 (40.6)
^b^PCL-C PTSD Indicator: *n* (%)	(*n* = 684)	
Yes (>34)	263 h(38.5)	19 (59.4)
No (<35)	416 (61.5)	13 (40.6)
^c^GAD Anxiety Indicator: *n* (%)	(*n* = 684)	
Yes (>9)	157 (23.0)	9 (28.1)
No (<10)	527 (77.0)	23 (71.9)

a Center for Epidemiologic Studies-Depression Scale Revised (Eaton et al., 2004).

b Posttraumatic Distress Disorder Checklist, Civilian (Blanchard et al., 1996).

c Generalized Anxiety Disorder Scale (Spitzer et al., 2006).

## Method

We sought to interview men who had experienced CLV. Lifetime cumulative violence was measured with the cumulative lifetime violence scale (CLVS-44) that includes 44 items that capture type (physical, psychological, sexual), timing (child or adulthood), focus (target or perpetrator), and context (e.g., family, community, workplace, intimate relationships, schools) of violence/abuse (Scott-Storey et al., 2020). Each Likert-type scale item includes questions about frequency from 1 (never) to 4 (often) and degree of distress from 1 (not at all) to 4 (very) (Scott-Storey et al., 2020). A severity score from 1 to 4 is obtained by summing and averaging frequency and distress scores, with higher scores indicating greater severity. We purposefully sampled variation in characteristics of interest such as severity of CLV (overall and by type, life stage, and form), demographics (e.g., age or geographic location), or a specific health problem. Those who identified in the survey that they were willing to be contacted for a follow up qualitative interview were invited to complete a one-on-one interview either in person, or by telephone, at a time and location of their choice. Nearly all men who were contacted for a follow up interview agreed to take part and, of those who refused, no pattern in demographic or other characteristics were identified. Following informed consent, interviews were completed in person or by telephone; men who chose to complete the telephone interviews identified that this was due to preference and/or geographical location.

Open-ended interview questions were aimed at allowing men to talk about their experiences of violence as a target and/or perpetrator, the ways these experiences affected their health, how they sought help, and how their experiences were influenced by their perceptions of gender (what it means to be a man). The following are examples of prompting questions during the interviews: “Tell me about your experiences with violence throughout your life.” “How has violence affected your health?” “How has what it means to be a man influenced these health effects?” Interviews took from 21 to 89 minutes and were audio-recorded and transcribed verbatim. We met regularly to discuss data collection and analysis so that the interview guide could be modified to explore emerging concepts and ideas. Participants were reassured that they could refuse if they preferred not to answer particular questions. Because of the sensitive nature of some study topics, debriefing materials including signs and symptoms of distress and local mental health resources were reviewed and given to all participants. To acknowledge the time and effort of participation, each man received a $25 honorarium. Using principles of data saturation as a basis for determining the nature of the sample, interviews were conducted with 32 men.

### Analysis

We used interpretive description to explore men’s mental health and CLV. Interpretive description is a non-categorical qualitative research approach that examines complexities of the human condition (Thorne et al., 1997). Consistent with the theoretical underpinnings of interpretive description, we approached the analysis with the understanding that reality is multifaceted and contextual and that knowledge is socially constructed between the researcher and participant (Thorne, 2008). The research question: “What are the mental health manifestations of CLV in men living in Eastern Canada?” was used to guide the data analysis. Early analysis in interpretive description involves an immersion of the data to derive units of meaning by coding the interview transcripts broadly (Thorne, 2008). Our initial coding resulted in large sections of data being named according to different forms of violence, health consequences, the ways that men dealt with health problems and gender.

Borrowing from grounded theory, interpretive description uses a constant comparative method (Thorne, 2008), an approach that informed our analysis by coding the data line-by-line with the aim to understand patterns of human behavior in a social context (Wuest, 2012). Codes were interpreted according to participants’ perspectives and the meaning they derived from their experiences (Thorne, 2008). We compared and contrasted codes to identify categories and patterns of behavior that men use to manage the negative mental health effects of CLV while also accounting for the influence of broader contextual factors. Constructing a typology (Glaser, 1978) was a useful mechanism for understanding how different intensity levels of mental health problems intersected with men’s subsequent responses. As analysis was underway, we met regularly to compare our interpretations of the emerging findings, clarify our interpretations of men’s perceptions, and refine our understanding of the meaning of the codes, concepts, and their relationships.

The analysis included a set of criteria to enhance rigor or trustworthiness as outlined by Lincoln and Guba (1985). Credibility was established through persistent observation or identifying which initial codes found during the beginning of the data gathering process remained during subsequent interviews. Transferability was demonstrated with dense descriptions of the data and explaining variation in the findings. Confirmability and dependability were addressed with the use of coded interviews, and research team discussions, memos, and an audit trail. Reflexivity also enhanced rigor through memoing that reflected insights derived throughout the analysis and how these related to our experiences, values, beliefs, and biases (Cutcliffe, 2003).

## Results

### Demographics

The average age of the participants was 43 (range 19–64) years and their diversity is evident in the descriptive profile in Table 2. CLV scores ranged from 1.23 to 2.81; all having reported physical, emotional, and/or sexual violence at home, school, within intimate partner relationships and/or in the workplace and community in their lifetimes; 28 (87.5%) having experienced CLV as both perpetrator AND target, 4 (12.5%) as target only, and none as a perpetrator only. Of the 32 men, seven had clinically significant scores on three mental health conditions, 10 on two conditions, and six on one condition (see Table 2).

**Table 2.** Descriptive Profile of Participants (*N* = 32).

Characteristics	*n* (%)
Cultural affiliation: *n* (%)	
Anglophone	29 (90.6)
Francophone	1 (3.1)
First Nations	1 (3.1)
Did not specify	1 (3.1)
Sexual identity: *n* (%)	
Heterosexual	27 (84.4)
Gay	1 (3.1)
Bisexual	3 (9.4)
Mood-based gender identity, Orientation fluid	1 (3.1)
Marital status: *n* (%)	
Single, never married	5 (15.6)
Married	17 (53.1)
Living with partner	7 (21.9)
Divorced or separated	3 (9.4)
Highest level of education: *n* (%)	
High School Diploma or less	5 (15.6)
Some Post-Secondary Education	13 (40.6)
College or University Degree/Diploma	14 (43.8)
Community size: *n* (%)	
Rural (<1,000)	10 (31.3)
Small town (1,000 to 29,999)	4 (12.5)
Medium city (30,000 to 99,999)	15 (46.8)
Large city (>100,000)	3 (9.4)
Employment: *n* (%)	
Employed	13 (40.6)
Unemployed	7 (21.9)
Disability, unable to work	3 (9.4)
Retired	4 (12.5)
Retired with some self-employment	2 (6.2)
Sick leave	1 (3.1)
Stay-at-home parent	1 (3.1)
Student	1 (3.1)
Total personal income past year (CAD): *n* (%)	
<$10,000	8 (25.0)
$10,000 to $24,999	8 (25.0)
$25,000 to $49,999	8 (25.0)
$50,000 to $74,999	5 (15.6)
$75,000 to $100,000	3 (9.4)

### Mental Health Effects of CLV in Men

The effects of CLV on men’s mental health are enacted through their relationships. CLV is a severing of mutuality with others, experienced as *detachment* or a disconnection from others. The mental health effect of *detachment* is *perceptual interference*, a cognitive disruption in the way men view the world that alters their self-worth, interpretation of how others see them, and their sense of security in relationships and interactions with others. *Perceptual interference* involves the belief that others pose a threat to their well-being and skepticism about being valued in their relationships, leading to overwhelming fear of being criticized or harmed that infiltrates their day-to-day lives. *Perceptual interference* exists within a context of social norms and gender role expectations to be a “real man” by being emotionally strong and independent, pressures that contradict men’s need for close attachments with others in the wake of CLV.

Men manage *perceptual interference* by *rectifying detachment*, a search to feel valued in their relationships and interactions. *Rectifying detachment* involves moving between engaging with others to meet emotional needs and disengaging from others to manage the psychological pain of low self-worth on their own. Attempts to *rectify detachment* occur through different strategies that are influenced by the intensity level of *perceptual interference*. All terms in italics are concepts that we identified from the data, representing the mental health outcomes of CLV in men.

### Perceptual Interference

The mental health outcome of CLV, *perceptual interference*, alters men’s interpretation of what is going on in their relationships and interactions with others. The term “*perceptual*” represents men’s interpretation of their environment and “*interference”* represents how CLV disrupts men’s thought processes and their capacity to connect to others. *Perceptual interference* involves a disruption of men’s sense of worth and well-being as they underestimate their value in relationships and misinterpret others’ intentions, believing that interactions with others are unsafe. *Perceptual interference* is a barrier to getting close to others and feeling secure interacting with people on a daily basis, leaving men feeling isolated and afraid.

**Figure 1.**
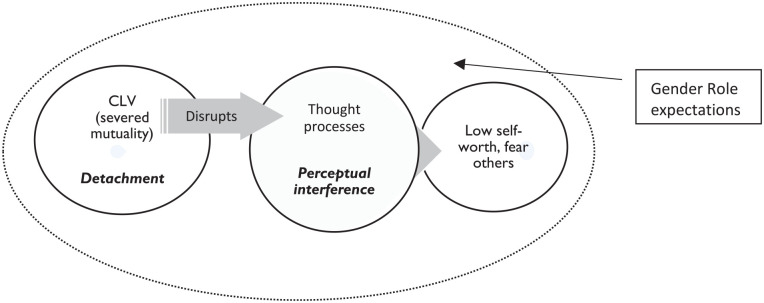
Perceptual interference: CLV disrupts thought processes, leading to low self-worth.

CLV leads to *perceptual interference* by generating *detachment* and the feeling of being cut off or separated from one’s relationships and the rest of the world. Men’s new and recurring experiences of violence as target and/or perpetrator in child and adulthood repeatedly sever connections and mutuality with others, undermining self-worth and giving rise to distrust and fear of harming or being harmed by others. Men’s capacity to function day-to-day is compromised due to overwhelming fear that persists even in times when violence is not predominant in their lives. *Perceptual interference* is fueled by gender role expectations to be a “man” including to be independent and strong, factors that discourage men from reaching out for help (Figure 1).

*Perceptual interference* is pervasive and reinforced by the normalization of men’s violence within family, workplace, and community contexts. Believing violence to be a natural part of being a man normalized violence for John: “If I wasn’t the man that I am I would . . .tend to turn away [from violence]. . . that’s what pretty much any woman would do, but I don’t. I turn toward it and I face it.” Facing potentially violent situations was his way of enacting gender by demonstrating strength through violence. Although John considered his willingness to face violence a strength, he acknowledged that it threatened his sense of safety. Intrusive thoughts that his life was in danger were a regular occurrence: “I’m always looking over my shoulder. . . to see if anybody is going to come stab me.” Fearing for his life led to pervasive feelings of anger, an experience shared by another participant, Samuel, who described losing his temper after he was almost hit by a car while walking. Samuel described that his anger was deeply embedded in his experiences as a target of childhood violence. He recounted how losing his temper made him ashamed of not living up to his father’s expectation to control his emotions:

I’ve never lost my temper and been violent with someone and looked back and said, “I’m glad I did that”, you know. Every single incident is involved with regret and I think I’ve seen that in my dad too. . . He used to always say [that] I shouldn’t have done that, you know. After a road rage thing, [dad would say] oh I shouldn’t have done that. I let it get to me.

Failing to meet his father’s standards to control Samuel’s anger made him feel less valuable as a man and as a person.

Importantly, *perceptual interference* is similar whether CLV is experienced as target, perpetrator or both. Violence as a target led to experiencing rejection and a fracturing of one’s sense of security altering men’s sense of self-worth and trust of others, contributing to the fear that they will continue to be targeted. Violence perpetration that impinges on another’s will and self-determination is a form of interpersonal separation that interferes with men’s capacity to create and maintain meaningful relationships and to have mutual reciprocal interactions with others. For example, perpetrating violence as a member of the armed forces led Jason to be “very suspicious of people” and to avoid his family, fearing that others would harm or reject him. Jason’s *perceptual interference* was intensified by gender role expectations as, “boys fighting was the norm.” He identified that an accumulation of lifetime violence experiences as both target and perpetrator lead to his *detachmen*t from others.

*Intensity levels of perceptual interference. Perceptual interference* has differing levels of intensity which are not determined by violence type or pattern, but rather men’s capacity to understand their CLV and the mental health outcomes. High levels of *perceptual interference* exist within a context of overwhelming distress from detachment and limited supports to help men gain insight into their needs. Lower levels of perceptual interference occurred during periods of time when violence was less prominent in men lives and they had time to gain insight into their CLV experiences and what they needed to make connections with others. Gender influenced perceptual interference intensity levels. For example, beliefs that aligned with more stereotypical gender norms and ideas that men ought to be strong and self-reliant increased *perceptual interference* among men by shaping their feelings about relationships and abilities to connect with others.

Felix’s low self-worth and fear of close relationships related to CLV as a child and adult intersected with the social norm that men are less capable of intimacy, contributing to a high level of perceptual interference that interfered with his ability to parent when his daughter was born.

Probably the main negative thing that came out of my life’s story with violence is the smothering over-protection of my children. . . Mothers bond pretty well straight away with their children. Fathers don’t, or at least this father didn’t and it took a while. . . for me to actually, like I loved her, but to actually feel a bond between us, it took a couple of months to do that and that’s when I started the downward spiral of overprotecting.

Felix unintentionally hurt his daughter with his efforts to protect her by trying to control her actions, emotions, and life decisions. High *perceptual interference* also led to avoiding help-seeking, which in turn further intensified *perceptual interference*. Limited access to formal and informal support to manage and address experiences of violence as target and/or perpetrator intensified *perceptual interference*, whereas availability of and access to support minimized disrupted thinking. Aurther explained how health professionals’ understanding helped decrease his *perceptual interference*:

[My family doctor] is very aware of what’s going on. . . she is concerned and she pays attention and she’s a wonderful doctor. . . I started developing some sort of awareness and started progressing into some sort of recovery from all that abuse and tried to develop some sort of self-identity that was positive and meaningful.

Developing awareness with the help of his family doctor helped to elevate Aurther’s self-worth. Men managed all levels of *perceptual interference* through a basic psycho-social process, *rectifying detachment*.

### Rectifying Detachment

*Rectifying detachment* represents men’s efforts to “fix” their sense of being disconnected from others with the goal of gaining self-worth in relationships and interactions with others. *Rectifying detachment* accounts for all behaviors and efforts to feel valuable and respected which can lead, in turn, to either closer relationships or further *detachment*. Tensions between competing demands to (a) rely on others to feel connected and have a sense of self-worth and (b) solve problems independently like a “man” are *rectified* by engaging or disengaging with others.

Engagement and disengagement can be either helpful or unhelpful depending upon the level of *perceptual interference*. Engagement in the context of low *perceptual interference* with less severe disruptions in thinking, involves learning about the negative consequences of violence including *detachment* and making efforts to improve their relationships and feel better about themselves. Disengagement in the context of low *perceptual interference* involves a greater understanding of CLV and *detachment*, which helped men to identify the factors that contributed to disruptive thinking. Recognizing how their relationships, interactions, and other situations led to fear, isolation, and low self-worth, men disengaged from these circumstances, and found new ways to connect with others. Alternatively, engagement in the context of high *perceptual interference* may involve perpetrating violence in an attempt to force others to value them by controlling them. Disengagement in the context of high *perceptual interference* involves moving away from others and isolating themselves in an attempt to avoid rejection.

**Figure 2.**
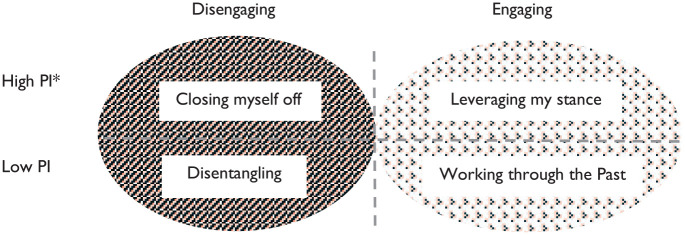
Rectifying detachment strategies. *PI = perceptual interference.

Strategies for *rectifying detachment* that vary according to the intersection of high or low *perceptual interference* and engagement or disengagement are: (1) *closing myself off*, (2) l*everaging my stance*, (3) *disentangling*, and (4) *working through the past* (Figure 2). The two former strategies are used under high perceptual interference and yield greater levels of detachment, whereas the latter two are used under low perceptual interference, yielding outcomes that are more helpful. Men choose strategies to fit their particular needs and circumstances at particular points in time with some using several different strategies and others relying on only one or two.

#### Closing myself off

In the context of high levels of *perceptual interference*, disengaging behavior involves *closing myself off.* This strategy is a way of hiding from others and shutting out the world by avoiding interactions or emotionally distancing oneself within a relationship. *Closing myself off* is used when men perceive that they have less power within their relationships and interactions and may be characterized by overwhelming feelings of fear, shame, and little hope for connections with others. *Closing myself off* is aimed at limiting harm by turning away from the shame and pain of low self-worth related to *detachment*. Fearing rejection, men hide emotions, and avoid close relationships and public places or social situations to reduce feelings of vulnerability and weakness. For most, *closing myself off* involved avoiding help seeking. Men described feelings of anxiety, stress, and worry about interacting with others, which made engaging difficult. Terance described how he handled his fear of being exposed and vulnerable around others by *closing myself off*:

I didn’t want to be noticed. I wasn’t able to communicate with people properly because I didn’t want to expose myself and I was always living in fear. . . I tried to hide and just stay in the background and I, I took jobs that would allow me to do that. So I worked mostly in the woods cutting wood. It would be like there was a lion after me or a bear after me, it was that bad. It just felt like a disaster was going to happen if I, if I had to speak in front of a couple of people or a group of people.

*Perceptual interference* lead Terance to falsely believing he was danger, limiting his capacity to engage in relationships. He interpreted his difficulty functioning as evidence he was not a “good solid man” due to his assumption that men are supposed to be: “stable, in control and calm.”

*Closing myself off* manifests in various ways. Feeling like a burden, some men purposefully avoided or withdrew from formal or informal resources and supports to avoid feelings of dependency, failure and weakness. Influenced by his father’s belief that getting help for emotional problems is a sign of being “crazy,” one man *closed himself off* by avoiding mental health professionals. *Closing myself off* may also occur unintentionally. Subtle forms of withdrawal may occur involuntarily to shift attention away from a perceived threat. For instance, one man found relief by “disassociating” as a way of *closing off* the stress of his girlfriend’s verbal abuse; others used alcohol and drugs to relieve feelings of worthlessness and *detachment*. Escaping painful feelings in this way was temporary and often worsened emotional problems. Men described feelings of depression, low mood, lack of motivation, low energy, guilt, and hopelessness, symptoms that exacerbated their withdrawal from life. Carl described, “I turned into like a vegetable and I wasn’t able to do anything, ahh, except for lay in bed.” Further, emotional pain sometimes escalated to unbearable levels generating thoughts about ending the pain through suicide that were deepened by feeling detached and loss of hope. Timothy explained, “It would be just easier if I didn’t have to deal with this, just get rid of myself.” Suicide is the most intense form of *closing myself off* as the intent is not only to decrease the feeling of *detachment*, but to put an end to the psychological pain of living.

#### Leveraging my stance

In the context of high levels of *perceptual interference*, engaging behavior involves *leveraging my stance* to get the advantage or upper hand within a relationship or interaction. Men exerted their will onto others to gain a greater sense of control, a pattern of behaviors men described as violence perpetration. *Leveraging my stance* is a strategy to manage *perceptual interference* associated with being targeted as well as that which results from perpetration. The goal is to elevate their position to one of power over others to establish self-worth. This strategy typically embodied high levels of anger as men believed that others were to blame for their relationship difficulties. In keeping with dominant gender role expectations, the only permissible emotion was anger, which was expressed on a continuum from frustration and irritability to rage. Desperate to connect with others while *leveraging my stance*, men placed their need to feel self-worth ahead of others’ needs. Having learned as a child to deal with negative feelings by being “tough,” Christopher who felt shame for being violent toward his wife, *leveraged his stance* with more abuse to avoid feeling weak.

I remember coming downstairs feeling so guilty and shamed and uh, I just felt almost sick with my [violent] behavior and pushed it all aside and mentally and physically abused her. It was a domination to bend somebody to my will.

Christopher referred to using “leverage” to force a connection with his wife by controlling her, positioning that allowed him to feel more valuable and powerful. Shane described dominating people whom he feared and who made him feel low about himself: “I’d always envy the people and of course I would belittle them to try to bring them down to my level or decrease the fear that I had of them.” *Leveraging my stance* was a way of managing social anxiety through an instant, albeit temporary, sense of self-worth.

*Leveraging my stance* for some men is a response to violence perpetration as well as victimization in a work context. For example, fighting in military combat may intensify *perceptual interference* and *detachment* that men manage by overpowering others. Living with post-war PTSD, Paul perceived that people from a certain race were dangerous and managed his fear and low self-value by further violence perpetration.

#### Disentangling

In the context of low levels of *perceptual interference*, disengaging behavior involves *disentangling* by stepping back from the things that disrupt men’s capacity to connect with others. *Disentangling* is possible when the acuity of CLV health effects are less intense as a result of receiving treatment for mental illness or time having passed since the violence exposure, permitting men the space and energy to evaluate the impact of CLV in their lives. Taking stock of factors contributing to their relationship fears and the reasons they feel isolated helps men to contemplate what is needed to prevent further *detachment*. In contrast to the increased isolation of *closing myself off, disentangling* involves disengaging from specific *detachment* behaviors and getting in touch with their emotions. Increasing insight into their relationships and understanding of the reality of their *detachment* counteracts disrupted thoughts and further decreases *perceptual interference. Disentangling* may begin by noticing how their actions influence *detachment*. After starting on medication for his anxiety problems, Nathan said he was better equipped to evaluate the impact of his aggressive behavior:

I would go from zero to a hundred, damage household stuff like walls, punch walls, stuff like that, kick barbeques. My wife would experience that violence and it would be bad. With the medication, it helped me be able to step back and slow down and watch my reaction and err, I don’t know how to explain it, but I could almost step outside my body and, and watch.

The medication helped Nathan too think through what was happening in the moment and could evaluate the consequences of his actions. This was not a self-blame exercise, rather, an opportunity to identify problems that contributed to *detachment*.

Examining the reasons for *detachment* helped men challenge social pressures that contributed to *perceptual interference*, for example, challenging the assumption that they are weak for needing help with mental health problems. Most participants identified that their father or a father figure’s difficulty expressing emotions, aggressive behavior toward the family, and expectations to fix problems by themselves influenced their capacity to develop attachments with others. Gaining insight in this way helped men to loosen the grip of these social norms. Edward elaborated:

Dad wasn’t very affectionate when I was younger. That shaped me in a way. A part of me still thinks that the man should be tough and quiet and it conflicts with the side of me that needs human contact.

*Disentangling* occurred through an awareness of how Edward’s father’s lack of affection influenced him as a child, helping him to loosen the grip of the masculine ideal to be tough and recognize his need for human contact.

*Working through the past*: In the context of low *perceptual interference* levels, men’s engaging behavior involves *working through the past*, a strategy of making changes toward gaining self-worth in their relationships based upon the insights that they glean sometimes through *disentangling. Working through the past* differs from *disentangling* in that men engage with trusted health care providers and family to examine and accept the negative impacts of CLV. Ultimately, this strategy involves changing unhelpful ways of relating to others, including the need to control others, setting boundaries in potentially harmful relationships, and feeling more comfortable being emotionally vulnerable with others. *Working through the past* exists within the context of others’ listening and understanding, strengthening men’s capacity to tackle the painful reality of *detachment. Working through the past* does not imply recovering from CLV. Rather, others’ understanding decreases *perceptual interference* and the belief that they have little worth, helping men to deconstruct assumptions that they ought to deal with their emotions independently. Instead of attributing their fear and loneliness to internal failure or weakness, *working through the past* is a process of expanding self-awareness and understanding how the environment and social factors shaped their identity.

Reflecting on experiences such as past abuse at home, feeling let down by teachers and other community members who missed the signs of men’s distress, the culture of violence at work, and gender role expectations in society, all helped men make sense of their problems. Comprehending that there were valid reasons for their mental health and relationship problems relieved feelings of weakness. Feeling less shame helped some men who had perpetrated violence find the strength to face how their actions hurt others and take action to change their behavior. For example, a therapist helped Daniel to recognize that being the target of childhood violence influenced his mistreatment of women: “I was misogynist . . . once I realized where my problems lay, I. . . fixed it. I acknowledged it. . . I even made the phone calls and made the apologies to some of the ladies.” Daniel believed that taking responsibility for his actions meant that he was being the man he always wanted to be.

*Working through the past* helped men let go of the belief that *detachment* was an indication of personal failure. Kevin explained his transition to seeking professional help: “It gets to a point where you’re like, I don’t really feel like doing this by myself anymore, when you relieve yourself and understand that it’s not weakness [to] reach out.” Increased self-worth motivated men to connect with others by being more assertive and asking for what they needed in relationships. Eric similarly sought help for low self-worth related to blaming himself for his experiences as a target of violence. He discovered that the violence interrupted his ability to trust others and that he had been pleasing others at the expense of his own needs:

Not having that sense of self until I really got into counselling and working through all those things and understanding it better and finding that identity. . . I have a much stronger sense of self and sense of who I am and what I deserve. . . I was able and am able to keep the identity of my core self together.

*Working through the past* helped Eric realize his worth. These different ways of *working through the past* did not solve men’s mental health and relationship difficulties but did help them gain confidence in making decisions and choices toward increasing their self-worth and capacity to connect with others.

## Discussion and Implications

Our purpose in this analysis of data from 32 men living in Eastern Canada was to investigate men’s perceptions of the relationship between their experiences of cumulative lifetime violence and their mental health. Our study findings advance traditional thinking of mental illness and violence in men through a gendered lens that accounts for men’s perspectives of their violence experiences as target and perpetrator throughout their lifetime. The extent to which mental health problems in men with CLV in this sample is manifested through their relations and interactions with others challenges the gender role expectation that men ought to be independent and the widely held assumption that men’s happiness is not reliant on their relationships (Yang & Girgus, 2019). An inductive qualitative approach allowed for a deeper contextual understanding of how men perceive the intersections of mental health and CLV severity, adding meaning to the significant correlations found in the full MVGHS sample. Importantly, our findings reflect the experiences of a diverse *community* sample of Eastern Canadian men in contrast to those of clinical or justice system samples common to many studies of men’s mental health or violence.

Another strength of this study is the focus on *cumulative* violence experiences as both a target and perpetrator, in childhood and adulthood, capturing a wide breadth of violence types (physical, sexual, psychological) in various contexts (e.g., family, intimate relationships, workplaces, school, community) that occur and recur in a lifetime. The finding that all men experienced *perceptual interference* and used *rectifying detachment* strategies in the context of being both a target and perpetrator of violence is critical evidence that the health effects of each of these forms of violence ought not to be examined separately. Mental health effects in this sample of men resulted from the severing of connections and mutuality with others implicit in experiences of violence as *both* target and perpetrator. Indeed, violence as target and as perpetrator is interrelated and shares a common etiology (Hamby & Grych, 2013).

Our findings about the interrelatedness between target and perpetrator are an important contribution to the conceptualization of violence in mental health treatment. Violence as a target may be privileged in mental health treatment because victims are viewed more worthy than perpetrators. Another common view is of violence as a unidimensional problem of mentally ill male perpetrators (Choe et al., 2008), caused by neurobiological factors (Siever & Rosell, 2016). The finding that both targeted and perpetrated violence kindled *perceptual interference* illuminates the entangled and complex cumulative experiences of violence in both forms and their impact on mental health. This new knowledge challenges health care providers to inquire about CLV history instead of focusing on the most recent or egregious violence experiences in the treatment of men with mental health problems. Approaches directed at addressing mental health problems in men must appreciate the intricacy of CLV and the interdependent relationship between violence as target and perpetrator. Our conceptualization that mental health is affected by CLV through detachment and subsequent *perceptual interference* offers direction rooted in men’s experiences.

Cognitive disruptions characteristic of *perceptual interference* are upheld by the mental health literature. Altered cognitive functioning previously has been identified in people living with depression who believe they are inherently defective, that the world places impossible demands on them, and that the future is bleak (Beck, 1979; Greenblatt-Kimron & Cohen, 2019). In the violence literature, *perceptual interference* is supported by Johnson’s (2013) finding that community violence “distorted” men’s capacity to process awareness of their reality, resulting in normalizing or growing “numb” to the violence in their day-to-day lives (p. 120). Ironson et al. (2019) found an association between targeted childhood sexual violence and cognitive disruptions including self-blame and negative thoughts about the world in men with PTSD, (Ironson et al., 2019). Importantly, cognitive disruptions accounted for men’s PTSD symptoms to a greater degree than the sexual violence itself. Notably, our qualitative evidence is additive, showing that CLV through detachment leads to *perceptual interference* that manifests in altered self-worth, fear of others and disruption of security in relationships with others.

The cognitive disruptions characteristic of *perceptual interference* identified in this study is supported by the mental health literature, including evidence that depression is associated with altered cognitive functioning. People living with depression believe they are inherently defective, that the world places impossible demands on them, and that the future is bleak (Beck, 1979; Greenblatt-Kimron & Cohen, 2019). The finding that CLV contributed to *perceptual interference* is supported by Johnson’s (2013) findings that community violence “distorted” men’s capacity to process awareness of their reality, resulting in normalizing or growing “numb” to the violence in their day-to-day lives (p. 120). The relevance of *perceptual interference* is demonstrated in a study that found an association between targeted childhood sexual violence and cognitive disruptions in men with PTSD, including self-blame and negative thoughts about the world (Ironson et al., 2019). Importantly, Ironson et al. (2019) found that cognitive disruptions accounted for men’s PTSD symptoms to a greater degree than the sexual violence itself.

*Detachment* as fueled by distrust of others in the wake of CLV is supported by the finding that childhood victimization of violence contributed to disrupted adult relationships in men who perpetrated IPV (Voith et al., 2020). Disrupted relationships and Detachment is influenced by gender expectations to be self-reliant, a challenge that reinforced men’s disengagement from others and reticence to seek help. This need for men to handle things on their own is in line with the pervasive view that masculinity is situated in an individualist context that, historically, has been synonymous with autonomy (Berger et al., 2013; Connell, 2005) and has been reported as a contributing factor to mental health difficulties in men (Herron et al., 2020; McCreary et al., 2020). Further, just as gender role expectations intensified social pressure to avoid close relationships for men in this study, Herron et al. (2020) found that pressure to be independent and emotionally detached from others contributed to mental health difficulties in men. McCreary et al. (2020) also found that depression in men was associated with gender role expectations to be self-reliant.

*Rectifying detachment* highlights the importance of emotional closeness and the extent to which men with CLV work to achieve a sense of self-worth within their relationships. The emotional needs identified by men in this study are in opposition to Western cultural assumptions that suggest that men ought to be rational or logical and displays of emotion imply weakness (Connell, 2005). In contrast, men in this study required insight and courage to identify their difficult emotions and gained confidence and strength when they addressed their feelings. *Rectifying detachment* demonstrates that despite having been disconnected from others in the wake of CLV, men in this study strived to connect and contribute to their relationships through both engagement and disengagement.

*Closing myself off*, a strategy of disengagement used when *perceptual interference* is high, is reminiscent of the use of substances, self-harm, and other behaviors to distract from, numb, or escape psychological pain (Briere, 2019; Levine, 2012). Our finding that low self-worth related to difficulty meeting gender role expectations evoked reluctance to seek help, another feature of *closing myself off*, is similar to avoidance of health care found in young men with mental health problems who fear appearing weak if they are not self-reliant (Cole & Ingram, 2020; Lynch et al., 2018). Mental health help-seeking and masculinities at the intersection of violence has received little attention. An exception is O’Donnell and MacIntosh (2016) who found that male targets of workplace bullying avoided help-seeking for mental health problems due to fear of appearing vulnerable. The current study extends these findings by illuminating that, despite feelings of fear and reluctance to seek help, men with CLV wanted others’ help. Understanding the paradox of men’s fears and desires for help might motivate service providers to engage men in gender-sensitive ways by asking about their CLV experiences (Drioli-Philips et al., 2020).

*Leveraging my stance*, a strategy of rectifying detachment through engagement, includes violence perpetration and challenges the unilateral narrative of victims being worthy of compassion and perpetrators undeserving of help (Hamby & Grych, 2013). Consistent with the intent to manage *perceptual interference* by seeking self-worth in their relationships, *leveraging my stance* is a way of engaging in relationships by exerting power or control over others. On the surface, this reinforces the view that men who perpetrate violence are “anti-social” or have a personality characterized by patterns of violating the rights of others (American Psychiatric Association, 2013). However, within the overall process of *rectifying detachment, leveraging my stance* is one way of connecting with others especially in times of high distress, shame or anger and is representative of a broader pattern of seeking to feel valuable within relationships. Perpetration in adulthood has been theorized as mimicry of having been the target of violence during children’s formative years (Orue et al., 2011). Although several men in this study believed that having been targeted in childhood contributed to perpetrating violence as adults, *leveraging my stance* demonstrates that violence perpetration is more complex and multifaceted. Understanding the underlying thoughts and intent of violence perpetration and difficulties relating to others in men with CLV might help service providers revamp punitive responses to men’s violence perpetration by integrating relationship building into their health care and helping men to achieve self-worth through non-violent means.

*Disentangling*, a strategy of rectifying detachment through disengagement when perceptual interference is low suggests that health care providers can help men to identify factors that contributed to *detachment* and distancing from people and events that contribute to feelings of low self-worth. Men *disentangled* by noticing contradictions between pressures to be stoic and independent versus their need to be emotionally connected to others, realizations that motivated further evaluation of their mental health. Other studies also found men with mental health problems struggled to be more caring and to have closer relationships in the face of gender expectations (Fogarty et al., 2015; Herron et al., 2020). Debunking the myths that men are unemotional, do not want to talk about their problems, or are indifferent about having close relationships is vital to helping men with CLV. *Disentangling* requires an easing of CLV intensity or a break from violence experiences; therefore, health care providers can help men to safeguard against relationships, workplaces, or community environments that place them at risk for violence. They can also help men access medical treatment of mood and thought problems that exacerbate violent perpetration.

*Working through the past*, a strategy of engagement during low perceptual interference, demonstrates men’s capacity to intentionally face the reality of their CLV and endure emotional vulnerability while exploring *perceptual interference* and psychological pain with trusted health care providers. While *working through the past* entails addressing violence that may have occurred earlier in men’s lives, findings from this study demonstrate the impact of ongoing violence and how it negatively influences men’s mental health through *perceptual interference* in the present. Understanding violence as an ongoing and cumulative experience is an important addition to the literature as researchers often focus on the impact of past violence or violence that occurred at distinct points in time (Scott-Storey, 2011). Efforts to manage *perceptual interference* parallel cognitive behavioral therapy, a talk therapy that aims to change negative moods by changing negative thoughts (Beck, 2016), as both aim to reduce interrupted thought processes. Although cognitive behavioral therapy is recognized as a best practice brief intervention for depression (Scogin et al., 2018; Wang et al., 2018), it may have limited effectiveness in addressing *perceptual interference*. The goal of cognitive behavioral therapy, to avoid overly-emotional states that override logical thinking and re-establish a sense of reality that is often determined by the therapist, may reinforce masculinity norms to be rational and unemotional that could leave men feeling blamed and/or shameful about their disrupted thinking and emotional difficulties.

Overall, our findings focusing on the impact of relationships and connections with others on the mental health of men with CLV are critical to creating services that are relational and grounded in making safe connections with others. Nurses have a vital role in fostering safe connections with men who have experienced CLV as the development of trust with clients, families, and the community is central to nursing care across a variety of settings. Developing a therapeutic rapport with men who have difficulty trusting others in the wake of CLV calls for a treatment lens informed by the negative impact of trauma and the broad factors that influence clients’ mental health difficulties. Trauma and violence informed care (TVIC) is a service delivery framework that acknowledges the negative impact of past as well as *ongoing* violence, social determinants of health, and how relationships with nurses impact health (Ponic et al., 2016). TVIC is an approach to service provision that recognizes the frequency and far-reaching impact of trauma and violence in people’s lives and aims to avoid re-traumatization in the delivery of care (Ponic et al., 2016). TVIC acknowledges that violence is rarely a static event that occurred in the past, but rather, is often an ongoing process that is sustained through structural and societal factors (Public Health Agency of Canada, 2018). Applied universally within the care environment, TVIC strengthens nurses’ capacity to promote health and reduce illness by recognizing the systemic barriers to men’s health including the stigma of having psychological problems and reaching out for help.

Primary care providers, who respond to the bulk of requests for mental health care (Talbot et al., 2014; Tovian, 2017), often narrowly target presenting symptoms (Georgaca, 2013). TVIC elevates nurses’ and other health providers’ awareness of how violence and social determinants of health including gender influence men’s mental health problems, capacity to support their well-being, and responses to service delivery. Understanding *perceptual interference* may decrease health care providers’ assumption that men are being “difficult”, resistant, or disinterested in their health when they avoid seeking help or do not engage in the treatment process. Knowledge that men with CLV are active in the promotion of their mental health by making efforts to connect with others and striving to feel valuable within their relationships may motivate health care providers to frame services around therapeutic relationships, peer connections, and family interventions.

Men have less difficulty accessing mental health services in safe spaces where they already meet, for example barbershops, workplaces, and sports settings with non-judgmental male peers (Harding & Fox, 2015; Robertson et al., 2018). Mental health services that acknowledge social pressures to be “manly” or stoic are essential to validating men and easing their sense of vulnerability as they seek help (Robertson et al., 2018; Schlichthorst et al., 2019). Veterans use peer-based models to support their members with mental health injuries that are based on sharing common experiences related to war violence, interventions that strengthen capacity to manage mental health problems in men with PTSD (Jain et al., 2016; Possemato et al., 2019). Still, specific mental health services for men that address CLV are rare, especially outside of large metropolitan areas. In all, men in this study were strongly influenced by other men’s viewpoints how to manage their emotions and relationships. Nurses can support men to form peer relationships with other men who have experienced CLV and challenge traditional gender norms that limit their capacity to develop healthy relationships.

## Conclusion

Interpretive description was a useful analytic approach for gaining a rich understanding of mental health and CLV grounded in men’s experiences. The strategies used by men to *rectify detachment* provide new insights for men themselves and health professionals and family members who seek to help them. The finding that relationships are central to men’s mental health in the wake of CLV is a call for an overhaul on service delivery for men within primary care, psychiatric institutions, and correctional facilities. A social determinants of health approach through an examination of CLV, gender, and relational factors are important considerations to supporting men’s mental health.

This study has limitations, including a convenience sample for the MVGHS. Another limitation is the absence of member checking. Conducting follow-up interviews with the participants for their feedback on the emerging findings might have strengthened the findings. While findings reflect the intended population of Eastern Canada, primarily living in New Brunswick, a bilingual province with a population of less than 1 million dwelling in rural areas and small cities, findings may be less applicable to men living in large metropolitan areas with other ethnic heritage. Nevertheless, the dominant cultures in our target population are Anglophone and Francophone and the majority of the population is white. As evident in Table 2, our sample reflects the diversity of Eastern Canadian men; for example, by marital status, sexual identity and socio-economic indicators. Study findings based on self-report is also a limitation. Future research could include expanding an investigation to these populations. Future research could also include viewpoints of men’s cumulative violence experiences and their mental health from family, friends, and service providers whose insight into men’s *perceptual interference* may add context to their distorted thought processes. Research evaluating services and interventions aimed at helping men manage the negative mental health effects of CLV is also needed.
